# Deregulation of *linc-PINT* in acute lymphoblastic leukemia is implicated in abnormal proliferation of leukemic cells

**DOI:** 10.18632/oncotarget.24401

**Published:** 2018-02-05

**Authors:** Andoni Garitano-Trojaola, Edurne San José-Enériz, Teresa Ezponda, Juan Pablo Unfried, Arantxa Carrasco-León, Nerea Razquin, Marina Barriocanal, Amaia Vilas-Zornoza, Bruno Sangro, Victor Segura, Felipe Prósper, Puri Fortes, Xabier Agirre

**Affiliations:** ^1^ Laboratory of Myeloproliferative Syndromes, Oncology Area, Foundation for Applied Medical Research, IDISNA, CIBERONC, University of Navarra, Pamplona, Spain; ^2^ Department of Gene Therapy and Hepatology, Foundation for Applied Medical Research, University of Navarra, Pamplona, Spain; ^3^ Liver Unit, CIBEREHD, Clínica Universidad de Navarra, University of Navarra, Pamplona, Spain; ^4^ Bioinformatics Unit, Foundation for Applied Medical Research, University of Navarra, Pamplona, Spain; ^5^ Hematology Service and Area of Cell Therapy, Clínica Universidad de Navarra, University of Navarra, Pamplona, Spain

**Keywords:** acute leukemia, lncRNA, *linc-PINT*, epigenetic, HMOX1

## Abstract

Long Non-Coding RNAs (lncRNAs) are functional RNAs longer than 200 nucleotides in length. Several lncRNAs are involved in cell proliferation and are deregulated in several human tumors. Few lncRNAs have been described to play a role in Acute Lymphoblastic Leukemia (ALL). In this study, we carried out a genome wide lncRNA expression profiling in ALL samples and peripheral blood samples obtained from healthy donors. We detected 43 lncRNAs that were aberrantly expressed in ALL. Interestingly, among them, *linc-PINT* showed a significant downregulation in T and B-ALL. Re-expression of *linc-PINT* in ALL cells induced inhibition of leukemic cell growth that was associated with apoptosis induction and cell cycle arrest in G_2_/M phase. *linc-PINT* induced the transcription of *HMOX1* which reduced the viability of ALL cells. Intriguingly, we observed that treatment with anti-tumoral epigenetic drugs like LBH-589 (Panobinostat) and Curcumin induced the expression of *linc-PINT* and *HMOX1* in ALL. These results indicate that the downregulation of *linc-PINT* plays a relevant role in the pathogenesis of ALL, and *linc-PINT* re-expression may be one of the mechanisms exerted by epigenetic drugs to reduce cell proliferation in ALL.

## INTRODUCTION

Acute Lymphoblastic Leukemia (ALL), the most common kind of cancer in children, is an hematological malignancy originated by un-controlled proliferation of B or T lymphoblast in peripheral blood, bone marrow and/or other organs [1]. ALL patients are characterized by aberrant genetic alterations such as chromosome translocations, mutations and deletions. These genetic insults disrupt the expression of oncogenes and tumor suppressor genes, resulting in the expansion of abnormal blasts. However, 40 % of ALL patients do not show such genomic alterations, suggesting that other mechanisms may be involved in the pathogenesis of this disease.

Transcriptome analysis by tiling arrays and RNA sequencing has led to the striking conclusion that while 70%–90% of the genome is transcribed, only 2% of the genome is dedicated to the transcription of protein coding genes [2]. Moreover, it has been shown that small non-coding RNAs such as miRNAs, play a relevant role regulating gene activity in cell homeostasis, differentiation and proliferation, and may also impact the diagnosis and prognosis tools for ALL and many other diseases [3–7]. In the past years, increasing number of studies have focused on the study of other RNA molecules such as long non-coding RNAs (lncRNAs) in ALL. This class of non-coding RNAs (ncRNAs) consists of RNA molecules longer than 200 nucleotides, which show low levels of expression and coding potential, and in most cases are cell-type and tissue specific [8, 9]. Most of the lncRNAs described to date modulate the expression of specific genes by guiding the chromatin remodeling factors, inducing DNA looping, regulating transcription, splicing, translation, post-translation modifications and mRNA stability [10–19]. All these functions play a relevant role in cell homeostasis and proliferation. In fact, several lncRNAs have been described as oncogenes or tumor suppressor genes in several human tumors [19]. Recently, Trimarchi T *et al* and Fang K *et al* described that *LUNAR1* and *ENST00000418618* lncRNAs are regulated by mutated *NOTCH1* and rearrange *MLL* in ALL patients, respectively, indicating that such lncRNAs may have oncogenic properties in this disease [20, 21].

In this study, we carried out a genome-wide expression analysis that shows that lncRNAs are deregulated in ALL, regardless of the genetic status of the disease. Specifically, we observe that the lncRNA *linc-PINT* (P53 Induced Noncoding Transcript) is downregulated in all the ALL cell lines and most B-ALL and T-ALL patients tested. Interestingly, *linc-PINT* re-expression reduces the proliferation of ALL cells. This effect could be mediated in part by Heme Oxygenase 1 (*HMOX1)*, whose expression also affects ALL cell-proliferation. Moreover, the re-expression of *linc-PINT* and *HMOX1* is observed upon treatment of ALL with epigenetic drugs, and therefore, it may be one of the molecular mechanisms induced by these drugs to cause anti-tumor effects in this disease.

## RESULTS

### LncRNAs are aberrantly expressed in ALL

**Figure 1:**
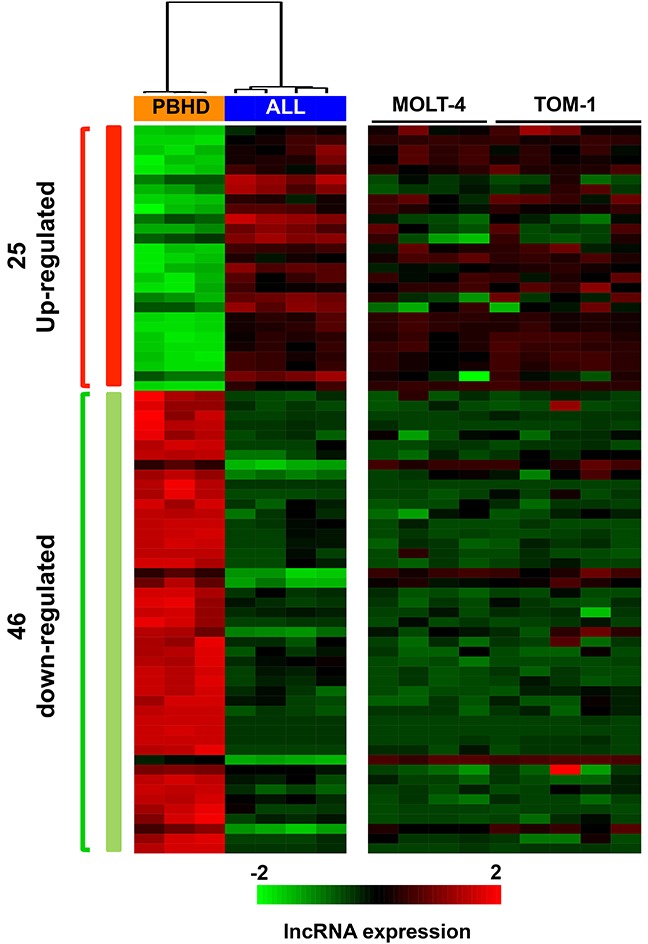
lncRNAs differentially expressed in ALL samples compared to healthy donor samples Hierarchical clustering using the differentially expressed lncRNAs between ALL patient samples and PBHD, including also the data obtained in TOM-1 and MOLT-4 cell lines. Red=overexpressed lncRNAs; Green= downregulated lncRNAs.

To analyze the expression of lncRNAs in ALL, we carried out a genome-wide lncRNA expression study using the Human SurePrint G3 microarray (Agilent, Santa Clara, CA), which evaluates the expression of 27958 Entrez genes and 7419 lncRNAs. We hybridized 4 primary ALL samples, 2 ALL cell lines and 3 peripheral blood samples obtained from healthy donors (PBHD). The normalized lncRNA array data was initially processed using an unsupervised principal component analysis (PCA) in which we observe that, similar to coding genes, the expression of lncRNAs shows a clear distinction between ALL primary samples and PBHD control samples (Supplementary Figure 1). We extended this first unsupervised analysis with a second supervised study to detect differentially expressed genes between primary ALL samples and PBHD samples. Analysis of the array by Ingenuity Pathway Analysis (IPA) showed that coding genes deregulated with a high statistical significance include genes associated with acute leukemia and cancer (data not shown). This served to validate our experiment design. A threshold of B>2 and fold change >1.5 was used to select 71 lncRNA probes that correspond to differentially expressed genes, 46 were downregulated and 25 upregulated in primary ALL samples (Figure 1, Supplementary Table 4). The downregulated or upregulated lncRNAs in primary ALL samples showed the same expression pattern (down or upregulated) in ALL cell lines MOLT-4 and TOM-1 (Figure 1). This indicates that these ALL cell lines represent a suitable model to study the role of the altered lncRNAs.

**Figure 2:**
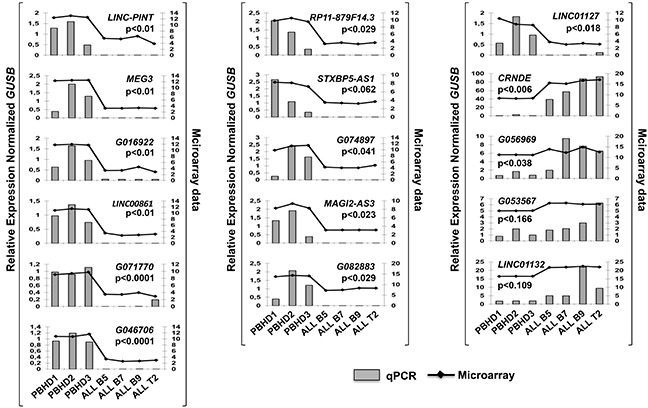
lncRNAs expression validation by Q-PCR Expression of 16 *lncRNAs*, including *linc-PINT* and *MEG3*, in ALL patients vs PBHD was measured with Q-PCR. *GUSB* levels were also quantified and used to calculate the relative expression (RE).

When the probe sequences were analyzed with the UCSC genome browser, we found that some probes matched the same lncRNA and few others were miss-annotated and hybridized to coding transcripts. Therefore, the 71 selected probes corresponded in fact to 43 lncRNA genes, 28 lncRNA genes down-regulated and 15 up-regulated.To validate these studies, 16 lncRNAs deregulated in ALL were selected, preferentially among those with higher scores, and their expression was analyzed by Q-PCR using the 4 primary ALL samples and 3 PBHD. The results show that 15 out of the 16 tested lncRNAs (93%) have the same expression pattern in the expression array (Figure 2). Globally, these results indicate that the expression of lncRNAs is clearly altered in ALL.

### *Linc-PINT* is deregulated in B and T-ALL

Among differentially expressed lncRNAs in ALL, we focused our study on those that had already been described in other human tumors but not in ALL, such as *CRNDE* (Colorectal Neoplasia Differentially Expressed), *MEG3* (Maternally Expressed 3) and *linc-PINT*. *CRNDE* was discarded at later stages of our studies because it has been recently described to encode a peptide [25]. Further, inhibition of *CRNDE* with siRNAs did not affect significantly the proliferation of MOLT-4 cells (data not shown).

**Figure 3:**
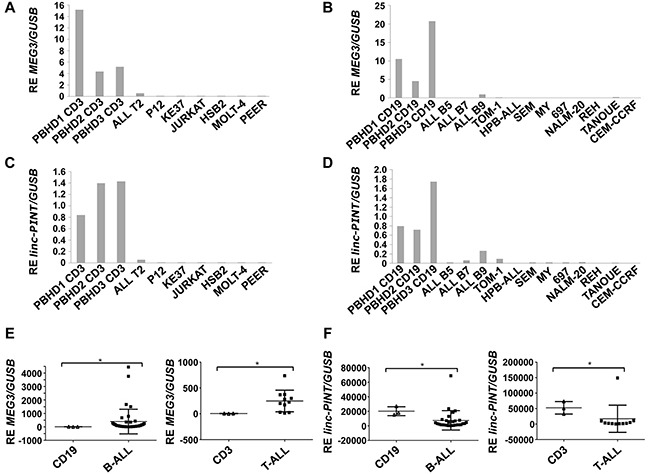
*MEG3* and *linc-PINT* are differentially expressed in ALL **(A and B)** *MEG3* expression in T and B-ALL cell lines. **(C and D)** *linc-PINT* expression in T and B-ALL cell lines. **(E)** *MEG3* expression in T and B-ALL respect to T (CD3+) or B (CD19+) lymphocytes obtained from healthy donors (^*^p<0.005 in T-ALL patients; ^*^p<0.0049 in B-ALL patients). **(F)** *Linc-PINT* expression in T and B-ALL respect to T (CD3+) or B (CD19+) lymphocytes obtained from healthy donors (^*^p<0.0385 in T-ALL patients: ^*^p<0.0214 in B-ALL patients). *GUSB* levels were also quantified and used to calculate the relative expression (RE).

To ascertain whether the expression of *MEG3* and *linc-PINT* was altered in ALLs derived from B or T lymphocytes, we analyzed their expression by Q-PCR in a different set of ALL-derived cell lines and we compared their expression with that of purified B cells from PBHD in the case of B-ALLs and purified T lymphocytes obtained from PBHD in the case of T-ALLs. This analysis showed decreased expression of the lncRNAs *MEG3* and *linc-PINT* in all B and T-ALL cell lines tested (Figure 3A–3D).

Once the down-regulation of *MEG3* and *linc-PINT* was confirmed in B and T-ALL cell lines, we evaluated their expression in 29 primary B-ALL and 11 primary T-ALL samples versus B or T lymphocytes purified from PBHD. As in ALL cell lines, the expression of *linc-PINT* was significantly decreased in primary B or T-ALL patient samples. However, we observed an increased expression of *MEG3* in primary B and T-ALL patient samples (Figure 3E and 3F). These results indicate that the expression of *linc-PINT* is down-regulated in ALL, suggesting that it could play a role in the pathogenesis of this disease.

### Overexpression of *linc-PINT* inhibits cell growth of ALL cells through apoptosis activation and cell cycle arrest

**Figure 4:**
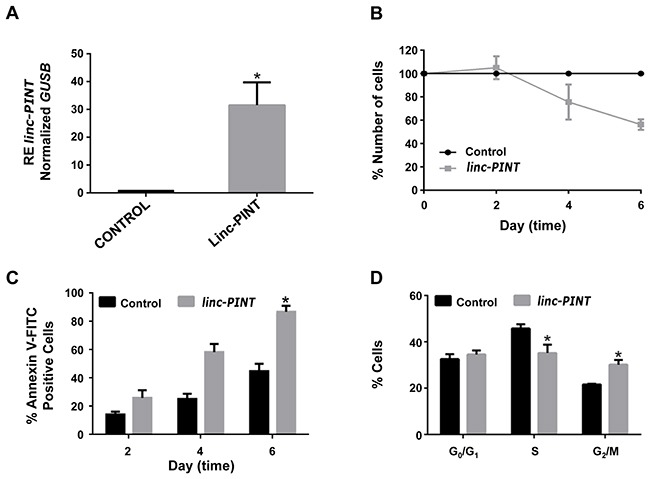
Overexpression of *linc-PINT* decreases MOLT-4 cell proliferation through apoptosis activation and cell cycle arrest at G_2_/M phase **(A)** *linc-PINT* expression after transfection of pcDNA3 plasmid that express this lncRNA (^*^p<0.003). **(B)** MOLT-4 cell proliferation after *linc-PINT* re-expression. **(C and D)** Apoptosis and cell cycle analysis after *linc-PINT* re-expression. Control: MOLT-4 cells transfected with an empty pcDNA3 plasmid. *linc-PINT*: MOLT-4 cells transfected with a pcDNA3 that expresses *linc-PINT*. *GUSB* levels were also quantified and used to calculate the relative expression (RE).

To define the functional role of *linc-PINT* in ALL cells, we re-expressed this lincRNA in ALL-derived MOLT-4. Transfection of cells with a plasmid harboring *linc-PINT* sequence produced a significant increase in the expression of this lncRNA (Figure 4A). Increased expression of *linc-PINT* resulted in a decrease in cell proliferation (Figure 4B) due to a significant induction of apoptosis (p<0.0005; Figure 4C) and cell cycle arrest at G_2_/M phase (p<0.01 for S phase and p<0.01 for G_2_ phase; Figure 4D). A drastic decrease in cell proliferation was also observed when *linc-PINT* was re-expressed in the B-ALL cell line My (Supplementary Figure 2). These results indicate that the deregulation of *linc-PINT* expression plays an important role in ALL cell proliferation.

### *Linc-PINT* activates *HMOX1* and their transcription is induced by Curcumin and LBH-589

**Figure 5:**
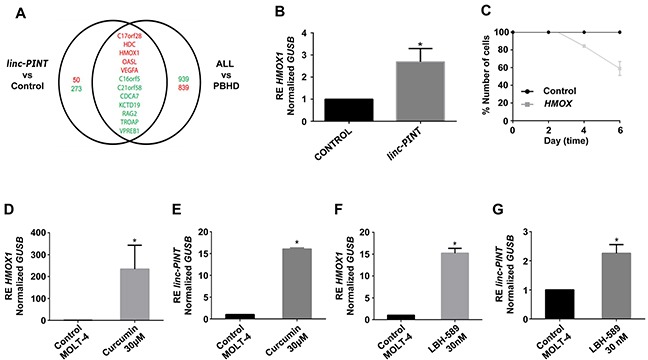
*linc-PINT* induces HMOX1 transcription and both genes are activated by Curcumin and LBH-589 in MOLT-4 cells **(A)** The Venn diagram shows the intersection between the differentially expressed genes in ALL vs PBHD (B>0) and in re-expressed *linc-PINT* vs control. Red numbers indicate upregulated while green numbers indicate downregulated genes. Red names: Genes down-regulated in ALL that were induced after *linc-PINT* re-expression. Green names: Genes up-regulated in ALL that were inhibited after *linc-PINT* re-expression. **(B)** *HMOX1* expression after *linc-PINT* re-expression (^*^p<0.03). **(C)** MOLT-4 cell proliferation after *HMOX1* re-expression. **(D)** *HMOX1* expression after treatment of MOLT-4 cells with the IC50 dose of Curcumin (30 μM) (^*^p< 0.004). **(E)** *linc-PINT* expression after treatment of MOLT-4 cells with the IC_50_ dose of Curcumin (30 μM) (^*^p<0.003). **(F)** *HMOX1* expression after treatment of MOLT-4 cells with the IC_50_ dose of LBH-589 (30 nM) (^*^p<0.01). **(G)** *linc-PINT* expression after treatment of MOLT-4 cells with the IC_50_ dose of LBH-589 (30 nM) (^*^p<0.01). *GUSB* levels were also quantified and used to calculate the relative expression (RE).

To examine the mechanism by which *linc-PINT* decreases the proliferation of ALL cells, we carried out a transcriptome analysis, using the Human SurePrint G3 microarray. The microarray was hybridized with RNA isolated from ALL derived MOLT-4 cells transfected with *linc-PINT* plasmid or from cells transfected with a control vector. In the analysis of the array, we identified 323 genes differentially expressed (50 genes up-regulated and 273 genes down-regulated; fold change >2 or <2) between cells with *linc-PINT* overexpression and control MOLT-4 cells. These results agree with a role of *linc-PINT* in silencing the expression of several genes, as has been described previously [26] (Figure 5A).

To identify genes that could mediate the anti-proliferative effect of *linc-PINT* in ALL, we searched for those genes deregulated in ALL cells versus PBHD (Figure 1) whose expression is also altered by *linc-PINT* overexpression (Figure 5A). In particular, we searched for genes downregulated in ALL cells (where *linc-PINT* expression is low) versus PBHD, that were also upregulated in ALL cells that overexpress *linc-PINT* versus control cells (Figure 5A). We also looked for the opposite scenario (genes upregulated in ALL cells and downregulated by *linc-PINT* overexpression). Only 12 genes fulfilled these criteria (Figure 5A). From these 12 genes, we focused on *HMOX1,* a gene up regulated after *linc-PINT* re-expression in ALL cells. We validated by Q*-*PCR the up-regulation of *HMOX1* after *linc-PINT* expression in MOLT-4 (p<0.03; Figure 5B) and My cells (p<0.04; Supplementary Figure 2C). As *linc-PINT* expression induces *HMOX1* and decreases cell proliferation, we wanted to evaluate whether *HMOX1* expression could also decrease proliferation of MOLT-4 cells and therefore, act as a mediator of *linc-PINT* effect. When we transfected ALL cells with a *HMOX1* expression vector, we observed that, similarly to *linc-PINT*, *HMOX1* overexpression reduced the proliferation of MOLT-4 cells (Figure 5C). This result is surprising as *HMOX1* has been described to have pro-tumoral effects.

Finally, we wanted to examine whether *linc-PINT* and *HMOX1* induction, and therefore inhibition of cell proliferation, are also observed when cells are treated with drugs with anti-tumoral properties for ALL and other malignancies. We choose the epigenetic drugs Curcumin and LBH-589, as we have previously shown that these drugs decrease ALL cell proliferation and induce apoptosis [28, 29]. Several molecular mechanisms have been described that explain the anti-tumoral function of these drugs. Interestingly, we observed that Curcumin and LBH-589 induce the expression of both *linc-PINT* and *HMOX1* in MOLT-4 and REH ALL cell lines (Figure 5D–5G and Supplementary Figure 3A–3D). Therefore, the induction of these factors may explain, at least in part, the anti-proliferative effects of Curcumin and LBH-589 in ALL.

## DISCUSSION

Over the last years, advances in transcriptome profiling have led to the identification of a large number of lncRNAs in normal and malignant tissues. In ALL, recent works have identified altered lncRNAs associated to common genetic alterations, such as translocations related with the 11q23 locus (t(4:11), t(11:19), t(9:11)), that alter the function of *MLL* gene in B-ALL, and *NOTCH1* mutations in T-ALL [20, 21, 30–32]. In the present work we observe that all ALL cells analysed, regardless of their genetic alterations, show a number of deregulated lncRNAs, suggesting that additional mechanisms may alter these elements in ALL.

Our transcriptomic analysis has identified 43 lncRNAs deregulated in ALL cells (Supplementary Table 4). We believe that most of them represent bona fide candidates. Out of the 16 chosen, the expression of 15 is concordant in the microarray and Q-PCR analyses (Figure 2). This represents a validation of 93% of the candidates. Among the 43 lncRNAs, *linc-PINT,* *MEG3* and *CRNDE* have been previously described to play a role in tumorigenesis. We believe that other candidates could play similar roles. In fact, analyses of the TCGA data shows that similarly to what we observe, *MAGI2-AS2* is significantly decreased more than 3 fold in bladder, breast, kidney and lung cancers, where it associates with survival, and *RP4-781K5.8* (*LINC01132*) is also increased in liver tumors and associates with survival of glioma, kidney and lung tumors.

*MEG3* is downregulated in several solid tumors (bladder, breast, cervical, colon, gastric, brain, lung, bone and prostate cancer) [33] and haematological malignancies like acute and chronic myeloid leukemias [34, 35] and it has been shown to exert tumor suppressor functions by inducing p53-dependent apoptosis [33]. Therefore, we were not surprised to find that *MEG3* was also decreased in ALL cells compared to controls (Figure 3A and 3B). However, *MEG3* shows a significant upregulation in B-ALL and T-ALL primary samples compared to control cells (Figure 3E). *MEG3* upregulation has also been observed in hepatoblastoma tissues compared to paired distant healthy tissues [36], suggesting that *MEG3* pro-apoptotic function could be counteracted in some tumors.

In the present work, we focus on the study of *linc-PINT*. We show that *linc-PINT* is downregulated in ALL cells and in most B-ALL and T-ALL primary samples compared to control cells (Figure 3C, and 3F). *Linc-PINT* downregulation has also been shown in colorectal tumors [26], in cholangiocarcinoma and in pancreatic cancer [37]. In the latter, it has been described that low levels of *linc-PINT* in the tumor correlate with poor prognosis after pancreatectomy while low levels of *linc-PINT* in plasma correlate with tumor recurrence [37]. These studies suggest that *linc-PINT* could be a tumor suppressor. In fact, we show that re-expression of *linc-PINT* decreases the proliferation rate of MOLT-4 cells due to induction of apoptosis and cell cycle arrest in G2 (Figure 4). The same effect was observed in other cancer cell lines, such as A549 (lung cancer) and HCT116 (colon cancer) cells [26]. Interestingly, *linc-PINT* re-expression in mouse embryonic fibroblasts (MEFs) promotes cell proliferation. As *linc-PINT* induces the opposite effect in MEFs and proliferating human cells, the biological role of *linc-PINT* may be cell-specific. Consequently, the molecular mechanisms that lead to *linc-PINT* expression and the pathways altered following its deregulation may vary among cell types. In MEFs, *linc-PINT* was reported to be induced by p53, to bind the Polycomb Repressive Complex 2 (PRC2) and to be necessary for its PRC2 targeting to the genome. Thus, in MEFs, *linc-PINT* mediates the trimethylation of H3K27me3 and consequently the silencing of specific genes [26]. Similarly, we find that re-expression of *linc-PINT* in MOLT-4 cells leads to the repression of several genes (Figure 5A). However, we also show that *linc-PINT* has a positive effect on gene expression and regulates PRC2 non-dependent genes, such as *C17orf28*, *HDC*, *OASL*, *VEGFA* and *HMOX1*.

*HMOX1* or heme oxygenase-1 is an inducible enzyme involved in heme degradation [38]. Carbon monoxide and other factors produced in the reaction, mediate *HMOX1* beneficial effects as antioxidant, anti-inflammatory, anti-apoptotic and pro-angiogenic. The protective effects of *HMOX1* are essential for tumor cell proliferation. Therefore, *HMOX1* is increased in several tumors (hepatoma, pancreatic and prostate cancers, among others) and can increase further in response to chemotherapy and irradiation [39]. Usually, pharmacological or genetic induction of *HMOX1* facilitates tumor progression while silencing *HMOX1* leads to decreased growth in culture cells and *in vivo* and has been suggested as an antitumor therapy. Surprisingly, in some breast, lung and prostate cancer cells high *HMOX1* expression correlates with decreased growth. Both overexpression and silencing of *HMOX1* in A549 cells lead to decreased proliferation, indicating that the levels of this factor may be critical. This is also observed in ALL cells. It has been described that inhibition of *HMOX1* in ALL cells decreases cell growth [40]. In this work we show for the first time that ALL cells also decrease growth after *HMOX1* overexpression. Further, we show that the same effect is observed after re-expression of *linc-PINT*, an *HMOX1* inducer, suggesting the possibility that *linc-PINT* could decrease ALL cell viability through *HMOX1* activation. Further experiments would be required to address whether this is indeed the case and to identify other factors that may also mediate the decrease in cell growth exerted by *linc-PINT*.

Previous studies have shown that the expression of *HMOX1* is promoted by the anti-tumoral drug curcumin in colon, prostate, breast and bladder cancers [41, 42]. Our previous work demonstrated that curcumin and LBH-589/panobinostat decrease ALL proliferation by activating cell death [28, 29]. In the present work, we observe that treatment with these epigenetic drugs leads to *linc-PINT* and *HMOX1* upregulation. Our results suggest that *linc-PINT*, and subsequently *HMOX1*, could mediate, at least in part, the anti-tumoral effect of these epigenetic drugs in ALL cells. Interestingly, curcumin and LBH-589/panobinostat induce *linc-PINT* expression also in healthy donor lymphocytes, but the fold increase is low compare to that observed in MOLT-4 cells (data not shown). This may reflect the fact that healthy donor lymphocytes express higher basal levels of *linc-PINT* than MOLT-4 cells and speaks in favor of the therapeutic use of epigenetic drugs.

In summary, in this work we demonstrate that several lncRNAs are de-regulated in T and B-ALL cells. Among downregulated lncRNAs, *linc-PINT* shows tumor suppressor properties. We propose that *linc-PINT* could reduce cell growth by upregulation of *HMOX1*. Moreover, we show that epigenetic drugs active in ALL, such as curcumin and panobinostat, induce *linc-PINT* and *HMOX1* expression and produce a decrease in ALL proliferation, suggesting that *linc-PINT* re-expression may be one of the mechanisms exerted by these epigenetic drugs to reduce cell proliferation in ALL. Collectively, this work demonstrates that the implication of lncRNAs in ALL is still underestimated, and suggests that the expression of lncRNAs could be more relevant for drug response than previously anticipated.

## MATERIALS AND METHODS

### Human samples and cell lines

B-ALL (TOM-1, 697, MY, NALM-20, SEM, REH, and TANOUE) and T-ALL (MOLT-4, HSB2, CEM-CCRF, JURKAT, KE37, HPB-ALL, P12-ICHIKAWA, PEER) derived cell lines were used for *in vitro* studies (Supplementary Table 1). Three primary B-ALL samples (ALL B5, B7 and B9) and one T-ALL sample (ALL T2) were also chosen as they show good proliferation after injection in immunosuppressed mice and, therefore, allow preclinical studies in animal models. Cell lines were maintained in culture in RPMI-1640 medium supplemented with 20% fetal bovine serum with 1% penicillin-streptomicin and 2% hepes at 37°C in a humid atmosphere containing 5% CO_2_. We used total peripheral blood samples (PBHD), purified T Lymphocytes (CD3^+^) and purified B lymphocytes (CD19^+^) obtained from healthy donors. CD3^+^ and CD19^+^ cells were selected by using CD3^+^ and CD19^+^ magnetic beads in AutoMACS device (Miltenyi Biotec, Cologne, Germany). In all cases cell purity was over 90%. Bone marrow mononuclear cells were obtained at diagnosis from patients with ALL (Supplementary Table 2) after an informed consent was signed by the patients or the patient’s guardians, in accordance with the Declaration of Helsinki. This study was approved by the Research Ethics Committee at the University of Navarra.

### Cell sorting, microarray hybridization and data analysis

Samples were processed using manufacturer protocols. Samples were hybridized to the Agilent SurePrint G3 Human Gene Expression 8×60K microarray [22]. Normalization of microarray data was performed using quantile algorithm (ALL samples=4, PBHD=3, TOM-1=5 and MOLT-4=4, *linc-PINT* re-expressed MOLT-4 sample=1, pCDNA3 MOLT-4 sample (Control=1). After quality assessment a filtering process was carried out to eliminate low expression probe sets prior to the analysis of expression differences between ALL and PBHD conditions. 38552 probe sets were selected for statistical analysis. R and Bioconductor were used for data processing and statistical analysis [23]. LIMMA (Linear Models for Microarray Data) was used to find out the probe sets that showed significant differential expression between experimental conditions (ALL vs PBHD) [24]. Genes were selected as significant using a B statistic cut off B>2.

To identify the mechanism by which *linc-PINT* decreases ALL cell viability, we used the same microarray expression mentioned previously. In this case, we hybridized *linc-PINT* re-expressed MOLT-4 cells and control cells (MOLT-4 transfected with pcDNA3 plasmid). As we wanted to analyze transcriptomic changes previous to proliferation reduction, neomycin was not used for selection of transfected cells. Instead, MOLT-4 cells were co-transfected with a plasmid which expresses *linc-PINT* (linc-PINT-pcDNA3) or with pcDNA3 and a plasmid that expresses GFP (pcDNA3-GFP) and GFP positive cells were selected by using cell sorting. We set up co-transfection conditions and used 1.5 μg/mL of each plasmid which correspond approximately to the same molar concentration. After 48 hours post-co-transfection, cells were centrifuged at 500 g for 5 minutes and the cellular pellet was resuspended in sorting buffer (phosphate-buffered saline (PBS without Ca/Mg++), 1mM EDTA, 25mM HEPES pH 7, 0.5% Fetal Bovine Serum (heat inactivated)). The GFP positives cells were sorted by using FACS canto (BD bioscience). The differentially expressed genes between *linc-PINT* re-expressed cells and control cells were selected using a log fold change threshold of 1.

### Quantitative real-time PCR (Q-PCR)

Total RNA was isolated from ALL cell lines and samples using UltraSpec reagent (Biotecs Laboratories, Inc) following manufacturer’s instructions. 1μg of total RNA was reverse transcribed using Superscript II RNAse H reverse transcriptase (Invitrogen). The quality of cDNA was checked by a multiplex PCR that amplifies Porphobilinogen Deaminase *(PBGD)*, Abelson Proto-Oncogene *(ABL)*, Breakpoint Cluster Region (*BCR)* and Beta-2-Microglobulin *(β2-MG)* genes. Q-PCR was performed in a 7300 Real-Time PCR System (Applied Biosystems), using 20 ng of cDNA in 2 μL, 1 μL of each primer at 20 μM, 6 μL of SYBR Green PCR Master Mix 2X (Applied Biosystems) in 12 μL reaction volume. Primer sequences are indicated in Supplementary Table 3. The following program conditions were applied for Q-RT-PCR running: 50 °C for 2 min, 95 °C for 60 s following by 40 cycles at 95 °C for 15 s and 60 °C for 60 s; melting program, one cycle at 95 °C for 15 s, 40 °C for 60 s and 95 °C for 15 s. All reactions were performed in triplicates. The relative expression of each gene was quantified by the Log 2^(-ΔΔCt)^ method using the gene Glucuronidase Beta *(GUSB)* as an endogenous control.

### Apoptosis and cell cycle analysis

For apoptosis assay, 100.000 cells were cultured at a density of 10^6^ cells/mL and incubated for 24 hours. The FITC Annexin V Apoptosis Detection Kit I (Cat. No. 556419, BD Pharmingen) was used following the manufacturer’s instructions, with some modification. Firstly, cells were washed twice with PBS and resuspended in 1X Binding Buffer at a concentration of 10^6^ cells/mL. 1 μL of FITC Annexin V (AV) antibody and 2 μL of propidium iodide (PI) were added and incubated for 15 min at RT in the dark. Finally, 400 μL of 1X Binding Buffer were added to each tube and analyzed by flow cytometry within 1 h using a FACScan flow cytometer (Becton Dickinson). Viable cells with intact membranes exclude PI, whereas the membranes of dead and damage cells are permeable to PI. The FITC AV staining precedes the loss of membrane integrity that accompanies the latest stages of cell death resulting from either apoptotic or necrotic processes. Therefore, cells that were AV-FITC-negative and PI-negative were classified as living cells, while AV-FITC-negative and PI-positive cells were classified as necrotic. AV-FITC-positive but PI negative cells were classified as early apoptotic as they present membrane integrity. Finally, AV-FITC-positive and PI-positive cells were classified as being in the end stage of apoptosis.

For cell cycle analysis, 250.000 cells were cultured at a density of 10^6^ cells/mL, washed twice with PBS and resuspended in 0.2% Tween-20 in PBS and 0.5 mg/mL RNAse A (Sigma) and incubated for 30 min at 37°C. Subsequently, cells were stained with 25 μg/ml of PI (Sigma) and analyzed using a BD FACScan flow cytometer (Becton Dickinson).

### Cell treatments

The HDAC inhibitor LBH589 (diluted in saline solution) (Novartis Pharmaceuticals, East Hanover, NJ, USA) and Curcumin (diluted in DMSO solution) (Sigma Aldrich Steinheim, Germany) were used. REH and MOLT-4 cell lines were treated with LBH589 and Curcumin at the half maximal inhibitory concentration (IC_50_) and maintained in culture for 2 days. Then cells were washed in PBS and used for different assays. The IC_50_ value was determined using GraphPad Prism log (inhibitor) vs response (variable slope) software (version 5, La Jolla, CA, USA).

### Cell viability assay

Cell viabilitywas analyzed after 48h of *in vitro* treatment or after 2, 4 or 6 days after *linc-PINT* nucleofection using the Celltiter 96 Aqueous One Solution Cell Proliferation Assay (Promega, Madison, WI, USA) following manufacturer’s instruction. All experiments were repeated three times.

### Plasmids transfection and neomycin selection

The linc-PINT-pCDNA3 and HMOX1-pCDNA3 plasmids express the transgene from a CMV promoter in a pcDNA3 background and have been described previously [26, 27]. For plasmids transfection we employed the nucleofection technique using an Amaxa system device (Amaxa GmbH, Koln, Germany). In different sets of experiments, the ALL-derived cell lines MOLT-4 and MY were nucleofected using 3 μg plasmid per 10^6^ cells in 100 μL of culture medium using the C-005 program and the Nucleofector device. Once cells were transfected, transfection efficiency and cell viability was analyzed by FACS Calibur (BD Biosciences). In MOLT-4 cells, the transfection efficiency was around 40% and cell viability 40-50%. Forty-eight hours post-transfection, Ficoll reagent (GE Healthcare, Pittsburg, PA) was used to remove dead cells. Remaining cells were washed twice with PBS 1X and neomycin 5 mg/mL (Gibco, Grand Island, NY) was used to select the transfected cells.

### Statistical analysis

Data are expressed as means ± standard deviation. A descriptive analysis was carried out to analyze the distribution of the samples. A logarithmic transformation was done prior to data normalization with a parametric test. Statistical significance between two samples was estimated with Student’s *t*-test. Differences were deemed significant for a real alpha of 0.05 (p<0.05). The statistical analysis was carried out by using R/Bioconductor.

## SUPPLEMENTARY MATERIALS FIGURES AND TABLES










