# Supplementary material for: Deregulation of linc-PINT in acute lymphoblastic leukemia is implicated in abnormal proliferation of leukemic cells
Source: Oncotarget. 2018 Feb 5;9(16):12842–52. doi: 10.18632/oncotarget.24401 (PMC5849178; doi:10.18632/oncotarget.24401)
Supplement: Supplementary file 1 [file oncotarget-09-12842-s001.pdf]

Deregulation of *linc-PINT* in acute lymphoblastic leukemia is implicated in abnormal proliferation of leukemic cells

SUPPLEMENTARY MATERIALS

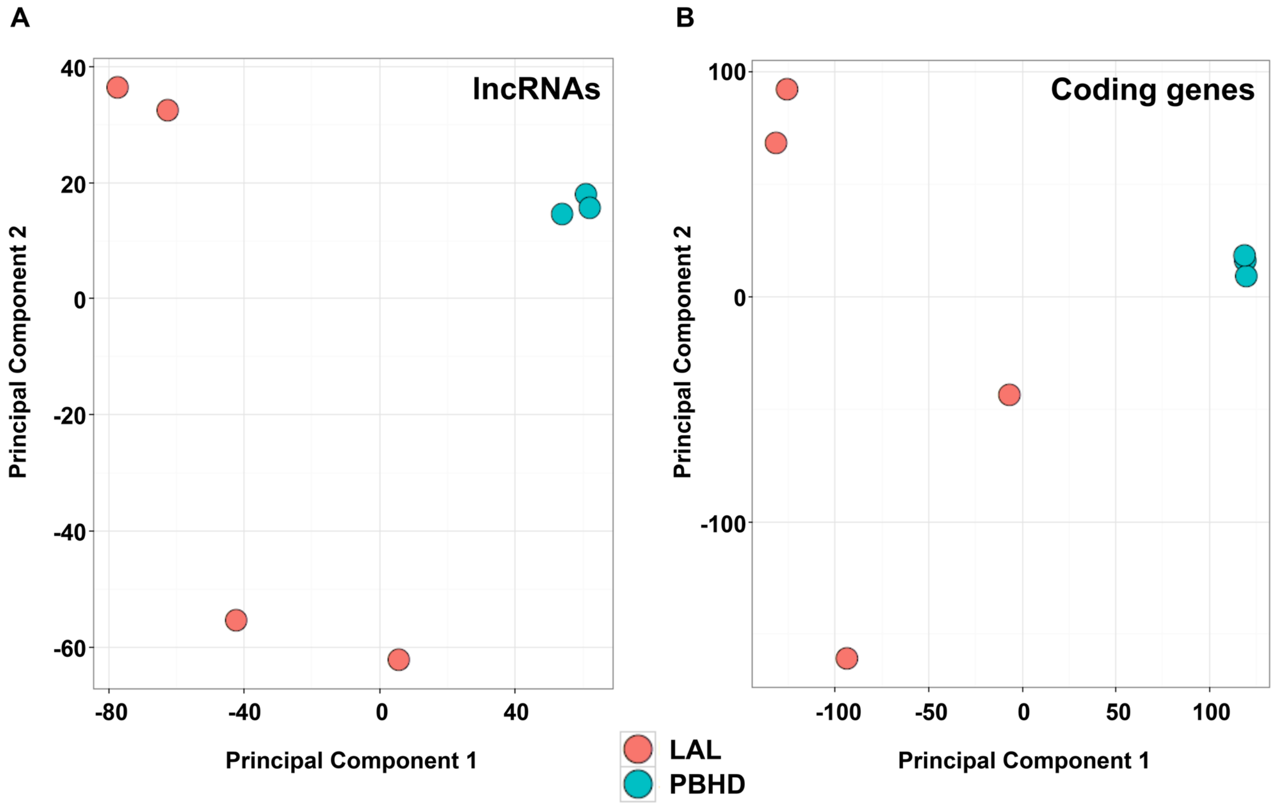

Supplementary Figure 1: Unsupervised Principal Component Analysis (PCA) in ALL patients and PBHD. Unsupervised PCA using the expression of lncRNAs **(A)** or mRNAs **(B)** detected in ALL patients and PCHD hybridized in microarray expression.

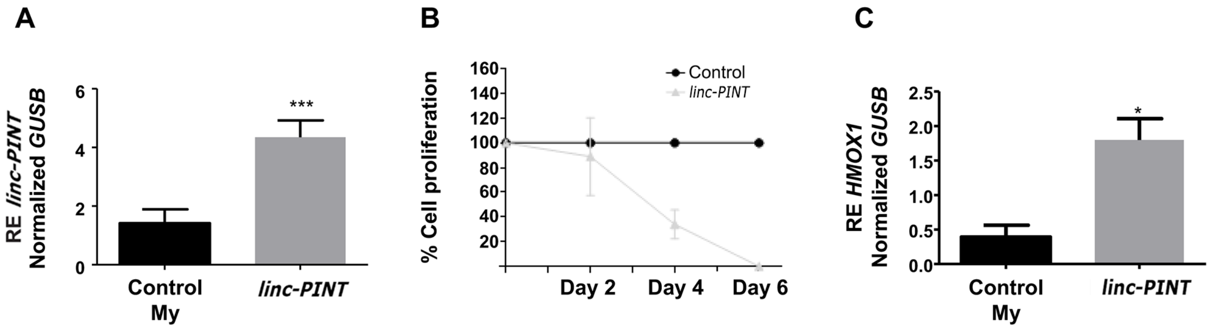

Supplementary Figure 2: Overexpression of *linc-PINT* decreases MY cell proliferation and increases *HMOX1* levels. **(A)** *linc-PINT* expression after transfection of MY cells with pcDNA3 plasmid that express this lncRNA ($^{***}p<0.0005$). **(B)** MY cell proliferation after *linc-PINT* re-expression. **(C)** *HMOX1* expression after *linc-PINT* re-expression ($^{*}p<0.04$). Control: MY cells transfected with an empty pcDNA3 plasmid. *linc-PINT*: MY cells transfected with a pcDNA3 that expresses *linc-PINT*. *GUSB* levels were also quantified and used to calculate the relative expression (RE).

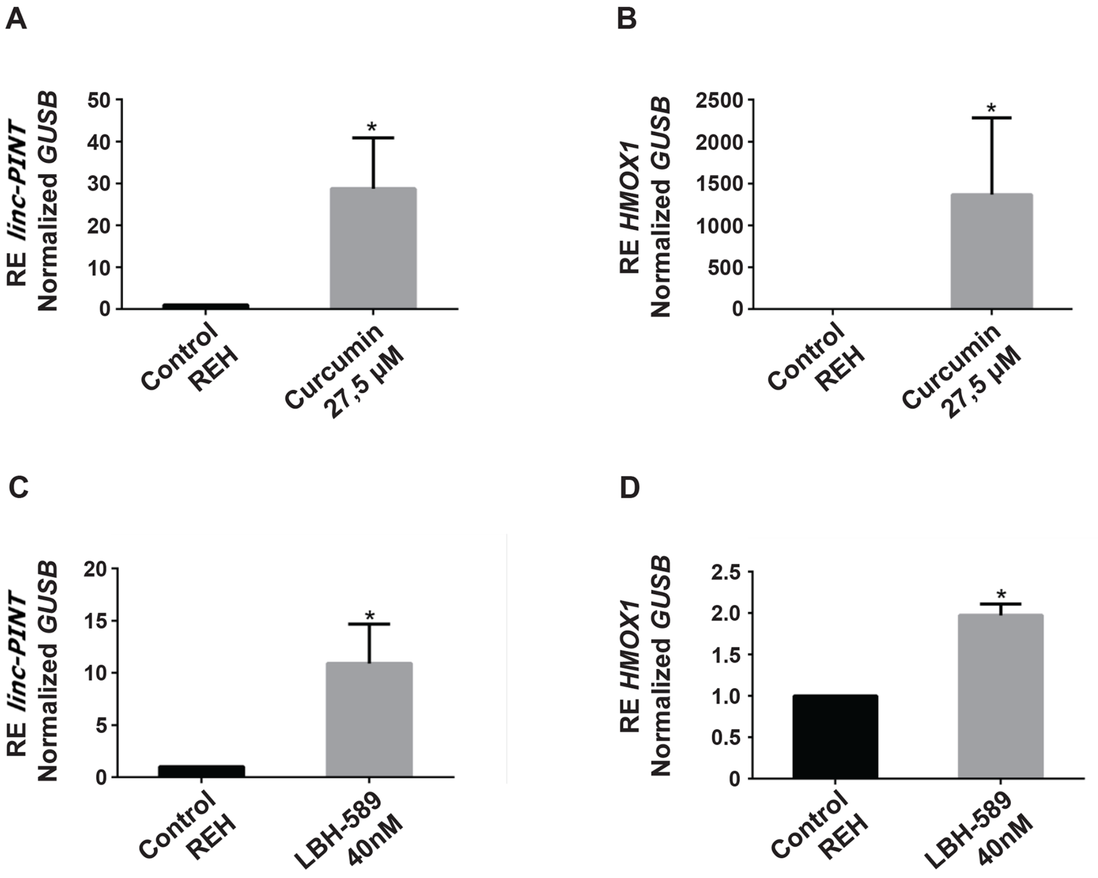

Supplementary Figure 3: *linc-PINT* and *HMOX1* transcription is activated by Curcumin and LBH-589 in REH cells. **(A)** *linc-PINT* expression after treatment of REH cells with the IC_{50} dose of Curcumin (27.5 μM). **(B)** *HMOX1* expression after treatment of REH cells with the IC_{50} dose of Curcumin (27.5 μM). **(C)** *linc-PINT* expression after treatment of REH cells with the IC_{50} dose of LBH-589 (27.5 μM). **(D)** *HMOX1* expression after treatment of REH cells with the IC_{50} dose of LBH-589 (27.5 μM). *GUSB* levels were also quantified and used to calculate the relative expression (RE).

Supplementary Table 1: Characteristics of all cell lines used in this study.

See Supplementary File 1

Supplementary Table 2: Characteristics of all primary patient samples used in this study. ND: No determined. +: positive for the analysis. −: negative for the analysis.

See Supplementary File 2

Supplementary Table 3: Oligonucleotides used in this study. Database used for gene names is indicated. G19, Encode; MiT, mitranscriptome.

See Supplementary File 3

Supplementary Table 4: List of lncRNAs deregulated in all. Validated candidates are highlighted. Database used for gene names is indicated. G19, Encode; MiT, mitranscriptome.

See Supplementary File 4
